# Post-puff respiration measures on smokers of different tar yield cigarettes

**DOI:** 10.1080/08958370802353443

**Published:** 2009-02-17

**Authors:** Frank K. St. Charles, George R. Krautter, Derek C. Mariner

**Affiliations:** 1 Brown & Williamson Tobacco Company, Macon, GA, USA; 2 R.J. Reynolds Tobacco, Winston-Salem, NC, USA; 3 British American Tobacco, Southampton, UK

## Abstract

The purpose of this study was to determine the effect of different tar yield cigarette brands on the post-puff inhalation/exhalation depth and duration for established smokers of the brands. The study was conducted with 74 established smokers of 1–17 mg Federal Trade Commission (FTC) tar products. The subjects were participating in a five-day inpatient clinical biomarker study during which time they were allowed to smoke their own brand of cigarette whenever they wished. On two separate days, the subjects' breathing pattern was measured using respiratory inductive plethysmography while they smoked one cigarette. This enabled the measurement of the post-puff inhalation volume, exhalation volume, inhalation duration, and exhalation duration for each subject after each puff on two of their own brand of cigarettes.

The subjects were grouped according to the FTC tar yield of their product: 1–3 mg; 4–6 mg; 7–13 mg; 14+ mg. The post-puff inhalation volume for the 4–6 mg group was significantly lower than both the 7–13 mg and 14+ mg groups, and the 4–6 mg group exhalation volume was significantly lower than the 14+ mg group (p < 0.05). No other differences were found at the 95% confidence level. When volumes were normalized to resting tidal volume (tidal ratio), there were no differences between the groups for any of the respiratory measures. No significant slope was found for correlations with FTC tar yield for inhalation volume (p = 0.11, mean = 833 mL, R = 0.19), inhalation tidal ratio (p = 0.93, mean = 1.73, R = −0.01) or lung exposure time (p = 0.92, mean = 4.1 s, R = −0.01).

## Introduction

Over the past few decades, marked shifts have taken place in the incidence of the different histologic types of lung cancer. The incidence of all lung carcinomas increased to a peak in US men in about 1990, since when it has declined, and to a plateau in US women in the late 1990s (American Cancer Society, 2008). During the period immediately preceding this, incidence of the most frequent distinct types, adenocarcinoma (AC) and small cell carcinoma, increased at a greater rate than squamous cell carcinoma (SCC) (Devesa et al., 1991) such that the ratio of AC:SCC changed from about 1:18 in 1950 to about 1:1.3 in 1995 (Wynder & Muscat, 1995). A similar shift from SCC to AC in esophageal cancer has been reported by Cockburn et al. (2005). The changes in lung cancer may be due in part to the apparent reduction in relative risk of SCC, but not AC, associated with lifelong filter cigarette smoking (Stellman et al., 1997). Medical advances in AC diagnoses over this time period are not considered to be driving this trend (Thun et al., 1997; Franceschi & Bidoli, 1999). A recent examination of SCC and AC data up to 2003 (Chen et al., 2007), however, shows that AC has also started to decline in both men and women.

The increase in AC has been attributed to changes in cigarette blend, design and smoking behavior (Wynder & Muscat, 1995; Hoffmann et al., 1996, 1997; Hoffmann & Hoffmann, 1997; Stellman et al., 1997; Thun et al., 1997; Franceschi & Bidoli, 1999; Cockburn et al., 2005). Higher nitrate levels in the blends of filter cigarettes, which could lead to higher amounts of nitrogen oxides and N-nitrosamines, especially 4-(methylnitrosamino)-(3-pyridyl)-1-butanone (NNK), in smoke, have been proposed as a contributing factor (Wynder & Muscat, 1995; Hoffmann et al., 1996, 1997; Hoffmann & Hoffmann, 1997). Higher levels of filtration and filter ventilation, and reduced tobacco weight have been used to reduce cigarette yields (Hoffmann et al., 1997). In addition, it has been found that as yield decreases, smokers tend to take larger and more frequent puffs (USDHHS, 1988; Scherer, 1999), and it may be that smoke is inhaled more deeply from low yield cigarettes than high yield cigarettes (Wynder & Muscat, 1995). Deeper inhalation would expose the more distal peripheral part of the lung, where most adenocarcinomas arise, to more smoke (Wynder & Muscat, 1995; Franceschi & Bidoli, 1999; Hoffmann et al., 1996, 1997; Hoffmann & Hoffmann, 1997; Stellman et al., 1997).

There have been a number of studies published which measured the inhalation patterns of smokers with cigarettes of differing tar and nicotine yield. Table 1 summarizes ten such studies published between 1978 and 1995, most of which were “switching” studies with small numbers of subjects. All but two of these switching studies showed no significant difference in inhalation patterns between cigarettes of differing yields. It is possible that a smoker's long-term habitual, post-puff inhalation pattern would not respond to these short-term switching tests. The switching study by Zacny and Stitzer (1988) did have each of the subjects smoking each of the five brands for five days/brand over a fiveweek period in a Latin Square design. This allowed some period for the possible alteration of inhalation patterns, yet the study still concluded “none of the respiration measures differed significantly across cigarette-yield conditions.” Two of the smaller scale studies found significant differences in inhalation patterns (Zacny et al., 1986; Woodson & Griffiths, 1992). Zacny et al. (1986) found an increase in lung exposure time when the ventilation holes of 1 mg tar products were blocked compared to fully open ventilation holes when the cigarettes were smoked without an orifice holder, but no significant difference when the cigarettes were smoked using an orifice holder. Woodson and Griffiths (1992) found a significant increase in inhalation volume when filtration was increased but draw resistance was held constant.

**Table 1:** Studies of post-puff inhalation patterns with smokers of differing yield products.

Reference	Method	N	Study type	Results
Guillerm & Radziszeski, 1978	Impedance pneumography	8	Switch - 31 mg to 16 mg tar cigarettes	NSD IV
Rawbone et al., 1978	Mercury strain gauge	15	Own brand with middle tar and low tar smokers	NSD IV
Tobin & Sackner, 1982	RIP	10	Switch - high tar unfiltered (26 mg) to low tar filtered (4 mg)	NSD IV, IT
McBride et al., 1984	RIP + nasal pitot tube	9	3 way Switch - Own brand, low (0.55mg) & high (0.90 mg) nicotine at 8 mg tar	NSD IV EV
Zacny et al., 1986	RIP	7	Switch med-high tar smokers to 1 mg tar; vents blocked 0, 50, & 100%, with & without orifice holder.	NSD IV. SD LET vents open < blocked. NSD IV, LET with holder.
Zacny & Stitzer, 1988	RIP	10	Switch - own brand + 4 differing nicotine brands	NSD IV, EV, LET
Woodson & Griffiths, 1992	RIP	17	Switch - own brand + own brand with differing filtration	SD IV ↓ with ↑ filtration
Robinson et al., 1992	RIP	5	Switch - same tar but change nicotine (0.6 versus 0.06 mg)	NSD IV, EV, IT ET lo nic > hi nic
Nil et al., 1986	Transthoracic impedance	117	Own brand and switch to 50% yield brand	Switch - NSD IV, EV, IV, EV Own - SD IV & EV ↑ as yield ↑
Hee et al., 1995	Inhalation index	108	Own brand	SD Inhalation index ↑ as yield ↓

RIP, Respiratory Inductive Plethysmography; NSD, no significant difference (p > 0.05); SD, significant difference (p ≤0.05); IV, inhalation volume; EV, exhalation volume; IT, inhalation time; ET, exhalation time; LET, lung exposure time.

Two studies report inhalation measures for large numbers of smokers smoking their own brand and give conflicting results. Nil et al. (1986) measured inhalation patterns on 117 smokers using a transthoracic impedance method. Measurements were made with the subjects smoking their own brand and a brand with 50% lower machine yield of nicotine. Results were grouped into five nicotine bands, with the bands determined by classifying the subjects into groups of equal size according to the nicotine yield of their usual brand. In comparing the effect of switching to the lower yield cigarettes, they reported “no significant changes for any of the measures of respiratory inhalation.” Inhalation volumes were found to be smaller for decreasing cigarette strength for the usual brand only; but this trend was not exhibited when smokers were switched to the lower yielding cigarettes. With the data separated according to gender, no trend was apparent with male smokers and the trend was magnified with female smokers. At the very least, these results did not show an increasing inhalation volume with increasing cigarette yield.

Hee et al. (1995) did not measure inhalation patterns directly but instead derived an inhalation index using carbon monoxide (CO), calculating the ratio (CO retained)/(CO produced) for 108 smokers. The retained CO was calculated from the carboxyhemoglobin boost produced from the first cigarette of the day and assumed standard values for CO capacity of hemoglobin, hemoglobin concentration, and blood volume. Results were grouped according to cigarette yield class with 36 subjects in each of three classes. The inhalation index was higher in males than in females and it increased with decreasing cigarette yield and with longer smoking experience. The inhalation index was evidently inferred to be a measure of inhalation depth since only the abstract stated that the inhalation depth increased with decreasing cigarette yield and with duration of smoking habit.

Therefore it is unclear whether smokers of lower yield cigarettes inhale smoke more deeply than smokers of higher yield cigarettes: one large scale study reports that lower yield products are inhaled less deeply, another large scale study reports that they are inhaled more deeply, and the less robust “switching” studies indicate that there is no difference. The present study was conducted in an attempt to clarify the issue.

## Method

### Study design

The clinical portion of this study was conducted by an independent contract research organization (Covance Clinical Research Unit Inc., Madison, WI, USA). The study was approved by Covance's Institutional Review Board and performed in accordance with applicable federal regulations. Subjects who participated in the study gave their informed consent, were told the purpose of the study and could with-draw from the study at any time. This was an independent study designed to utilize smokers that were participating in a separate biomarker correlation study (St. Charles et al., 2006). Habitual smokers were recruited through Covance's internal subject database or local advertisements. Enrollment criteria included men or non-pregnant, non-lactating women, between 21 and 65 years of age, who smoked at least 15 cigarettes a day of the same cigarette brand during the previous year. Use of any cigarette brand other than subject's declared own brand, any other alternative tobacco product, or use of any nicotine replacement therapy (gum, patch, tablets) during the study period was prohibited.

The number of qualified subjects assigned into one of four Federal Trade Commission (FTC) Nicotine Free Dry Particulate Matter (NFDPM or “tar”) yield range groups (mg tar yield range per cigarette) were: 15 low ultra (ULL; 1–3 mg), 20 high ultra (ULH; 4–6 mg), 20 lights (LTS; 7–12 mg), and 19 full flavor (FF; 13 mg+). These designated tar yield range groups span the range of FTC tar yields found in commercially available US filtered cigarettes. The recruitment goal was to enroll 20 smokers per tar range group; however, even with additional recruitment attempts, only 15 smokers enrolled in the ULL group (market share < 2%). One subject in the FF group withdrew from the study due to illness and the attendant data was not used. Table 2 summarizes the subject demographics and brand characteristics by band.

**Table 2:** Subject demographics and cigarette brand characteristics by tar band.

	ULL	ULH	LTS	FF	Total
Subjects					
Number	15	20	20	19	74
Gender (M/F)	8/7	12/8	10/10	14/5	44/30
Height cm	173 (159–204)	174 (157–194)	174 (161–187)	177 (165–190)	175 (157–204)
Weight kg	77 (57–104)	75 (42–98)	78 (56–98)	77 (60–100)	77 (42–104)
Age	35 (21–54)	32 (21–47)	34 (21–64)	32 (21–53)	33 (21–64)
Menthol smokers	3	3	5	7	18
Brands					
Menthol/non-menthol	2/5	3/6	4/8	4/6	13/25
Tar (mg/cig)	1.4 (0.7)	5.4 (0.5)	10.1 (0.9)	15.0 (1.7)	8.3 (5.1)
Nicotine (mg/cig)	0.15 (0.05)	0.46 (0.04)	0.77 (0.06)	1.07 (0.14)	0.64 (0.34)
CO (mg/cig)	1.9 (0.9)	6.8 (0.6)	11.6 (1.4)	13.9 (2.0)	8.9 (4.6)
Puffs/cig	7.6 (0.7)	8.0 (1.1)	7.9 (1.0)	8.2 (0.9)	7.9 (1.0)

Height, weight, and age are shown as the mean (range). Cigarette yields are shown as the mean (standard deviation) for the FTC smoking method.

Subjects were confined to the clinic for a six-day period. Confinement periods were staggered and limited to approximately ten subjects (generally of the same tar range group) at a time. During confinement, subjects smoked their usual cigarette brand ad libitum in a dedicated smoking room equipped with ventilation and air filtration. Smoking behavior measurements of post-puff inhalation volume and duration, exhalation volume and duration, and tidal volume were taken twice from each subject in two separate sessions with at least one day separating each session.

### Apparatus

Respiration patterns were measured using a Respitrace® 204 respiratory inductive plethysmograph connected to a portable computer running RespiEvents™ 5.2 software (Non-Invasive Monitoring Systems, Miami Beach, FL). This device measures inhalation patterns non-invasively by use of RespiBand Plus respiration bands (SensorMedics Corp., Yorba Linda, CA) around the chest and abdomen. The bands are made of very weak elastic so that they do not exert a noticeable pressure or interfere with breathing patterns.

**Figure 1:**
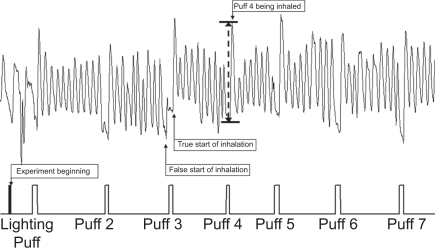
Respitrace pattern: tidal volume versus time (upper) with event marker (lower).

Figure 1 shows a Respitrace pattern for a smoking session. Post-puff breathing cycles were identified using an event marker connected to one of the analog inputs on the Respitrace. A simple circuit with a push button switch, a 1.5 V battery, and a potentiometer to limit voltage to less than 1.0 V provided the signal. The button was pressed when the subject put the cigarette to their lips and was released when the cigarette was removed. In addition, three rapid pulses were used to mark the beginning of an experiment. The event marker pulse was recorded and displayed simultaneously with the breathing pattern traces.

The RespiEvents functions for inhalation volume (ViVol), exhalation volume (VeVol), inhalation time (Ti) and exhalation time (Te) were used to measure the majority of the postpuff waveforms. The data were saved to an ASCII file on a breath-by-breath basis. The raw data for time, tidal volume (Vt), and signal from the event marker were saved to a separate ASCII file. Occasionally, the software would detect a false start of inhalation (Figure 1). These were identified by a brief minimum in signal slightly before or at the beginning of the event marker signal. For these cases the Vt value for the trough and peak of the waveform, along with the respective times, would be read from the ASCII file of the raw data. By subtracting the value at the true beginning from the value at the peak, the correct value for the inhalation time and volume was obtained.

### Procedure

Measurement sessions were scheduled in the afternoon. Each subject smoked one cigarette of their own brand during a session. Subjects were seated on a stool with no back to prevent interference with the bands. A nurse was present to ensure proper placement of the Respibands, to record the data, and to press the event marker for each puff. Subjects were not allowed to view their Respitrace patterns during the experiment.

Two types of calibration were used. When the Respitrace was turned on, a 5-min automatic calibration measured the normal breathing pattern of the subject. Breathing volumes were then automatically reported relative to the mean resting volume. The cigarette was not lit until this initial calibration was completed. Immediately following the cigarette, inhalation volume was further calibrated using an 800 mL Spirobag (Ambulatory Monitoring Inc., Ardsley, NY) by having the subject re-breathe at least six times into the Spirobag, take a brief rest, and then re-breathe another six breaths into the Spirobag. The initial inhalation breath was not used since it was not taken from the Spirobag, so this gave ten calibration breaths per session.

## Results

All puffs from both replicates for each subject were averaged to give a mean value per subject. The means, standard deviations, and ANOVA were calculated using the mean value per subject. This approach was chosen for two reasons. Calculating the values for all puffs combined would give more weight to the subjects with the greatest number of puffs per cigarette and less weight to the subjects with the fewest number of puffs. In addition, using the mean value per subject avoids the problem of multiplicity when making multiple measurements on the same subject (Altman & Bland, 1997).

The mean values and standard deviation for all respiratory measures are given in Table 3. Inhalation and exhalation volumes are reported on an absolute basis (mL) and also as a proportion of the resting tidal volume (inhalation or exhalation tidal ratio) to compare with their normal resting breathing pattern (McBride et al., 1984). Also tabulated are the p-values for a single factor analysis of variance (ANOVA) with tar band being the single factor. None of the measures gave a significant effect due to tar band. The effect of tar band on inhalation volume approached significance with a p-value of 0.09, however, when expressed as the inhalation tidal ratio, the p-value increased to 0.51. Notice that the LTS and FF bands had both the highest inhalation volume and resting tidal volume. Using Duncan's multiple range test, the post-puff inhalation volume for the ULH group was significantly lower than both the LTS and FF groups and their exhalation volume was significantly lower than the FF group (p < 0.05). No other differences were found at the 95% confidence level. When tidal volume was taken into account (using the inhalation tidal ratio) there were no differences between the groups for any of the respiratory measures. It should be noted, however, that the study was somewhat underpowered for proper evaluation of Type II errors; with a probability for the inhalation volume measurement of 0.55 (based on the group size of 20) and a minimum sample size for β = 0.2 of 42.

**Table 3:** Mean (SD) post-puff respiratory measures by tar band and for all subjects combined.

	ANOVA p-value	ULL	ULH	LTS	FF	All
Volume (mL)						
Inhalation	0.09	778 (240)^a,b^	731 (290)^a^	936 (280)^b^	876 (270)^b^	833 (279)
Exhalation	0.20	841 (259)^cd^	799 (200)^c^	976 (419)^cd^	960 (285)^d^	897 (308)
Resting tidal volume	0.15	443 (121)	482 (132)	555 (192)	509 (115)	500 (148)
Tidal ratio		^a^	^a^	^a^	^a^	
Inhalation	0.51	1.84 (0.67)	1.56 (0.52)	1.78 (0.65)	1.75 (0.50)	1.73 (0.58)
Exhalation	0.65	1.97 (0.59)	1.75 (0.49)	1.82 (0.68)	1.94 (0.54)	1.86 (0.58)
Time (sec)		^a^	^a^	^a^	^a^	
Inhalation	0.80	2.04 (1.92)	1.77 (0.81)	1.66 (0.77)	1.87 (1.07)	1.82 (1.16)
Exhalation	0.35	2.27 (0.98)	2.00 (0.52)	2.47 (1.04)	2.40 (0.85)	2.28 (0.87)
Total lung exposure	0.73	4.31 (2.22)	3.77 (0.95)	4.11 (1.44)	4.27 (1.76)	4.10 (1.59)
Other data						
Puffs taken	0.09	12.0 (3.0)	13.0 (3.1)	11.9 (2.7)	10.6 (3.0)	11.9 (3.0)

ANOVA p-values are for effect of tar band. Measures with the same letter are not significantly different at the 95% confidence interval using Duncan's multiple range test.

**Figure 2:**
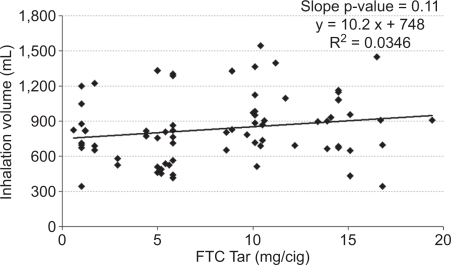
Mean inhalation volume per subject versus the FTC tar yield of their brand.

**Figure 3:**
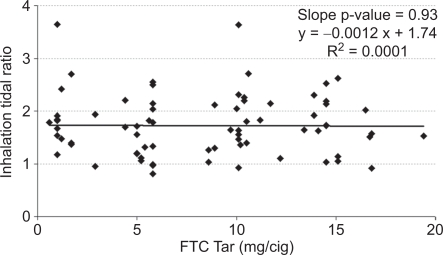
Mean inhalation tidal ratio per subject versus the FTC yield of their brand.

**Figure 4:**
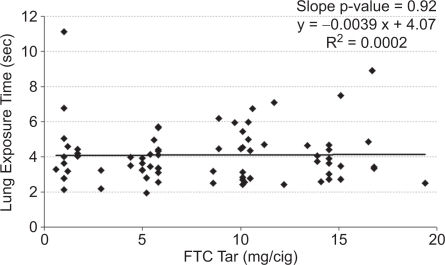
Mean lung exposure time per subject versus the FTC tar yield of their brand.

It is possible that an inappropriate choice of tar band could have masked differences. Therefore, the mean values for each subject were plotted versus FTC tar yield for inhalation volume (Figure 2), inhalation tidal ratio (Figure 3), and total lung exposure time (Figure 4) along with a linear regression line for each. None of the measures correlated significantly with the tar yield at the 95% confidence level. Inhalation volume versus FTC tar showed the highest level of significance (Figure 2, slope p = 0.11), and in this case the slope was positive. But any hint of a correlation of inhalation volume disappeared when normalized to the subject's resting tidal volume (Figure 3, slope p = 0.93).

Respiration measures were also compared between menthol and non-menthol smokers. The mean (standard deviation) inhalation tidal ratio was 1.52 (0.47) for menthol smokers and 1.79 (0.60) for the non-menthol smokers. A two sample t-test assuming unequal variance gave a p-value of 0.054 which borders on significance. Mean (SD) inhalation volumes were 753 (217) mL and 859 (294) mL (p = 0.11) and total lung exposure times were 4.0 (1.4) sec and 4.1 (1.7) sec (p = 0.85) for menthol and non-menthol smokers, respectively.

## Discussion

Overall, post-puff ventilation measures were very similar to those measured in other studies (Tobin et al., 1982; Tobin & Sackner, 1982; Zacny et al., 1986; Zacny & Stitzer, 1988) with mean inhalation volumes reported from 649 to 841 mL, mean lung exposure times of 4.5 to 5.6 s, and a mean inhalation tidal ratio of 1.77. In addition, post-puff exhalation volumes have been reported to be slightly greater than inhalation volumes as was found in this study (Nil et al., 1986; Zacny & Stitzer, 1988). However, Nil et al. (1986) reported post-puff inhalation volumes to be the same as tidal inspiration volumes (i.e., inhalation tidal ratio = 1.0) of 0.5 L for men and 0.4 L for women while McBride et al. (1984) reported an inhalation tidal ratio of 1.45.

The results from this study contradict those of Hee et al. (1995) in that no trend was observed in any respiration measure with cigarette yield. Hee et al. (1995) reported that inhalation depth increases with decreasing cigarette yield; however, because inhalation depth was not directly measured, we interpret this to mean the authors inferred inhalation depth from their inhalation index. The inhalation index was a calculated ratio between the amount of CO retained to the amount of CO produced by the cigarette. The inhalation index for the lowest yield cigarette (mean 3.4 mg tar) was reported to be significantly higher than that of the other two cigarette classes (means of 9.0 and 14.7 mg tar). However, Zacny et al. (1987) showed in two separate experiments that carbon monoxide boost had little if any relationship to inhalation volume. One experiment had subjects inhale at 0, 20, 40 and 60% of vital capacity with a 4-s breath hold time and another had subjects inhale at 0, 10, 20, 40, and 60% of vital capacity with no breath hold time. No CO boost was found with no inhalation (0%), but within-experiment CO boost was very similar (and showed no trends) with any of the other inhalation depths tested. It appears that the inhalation index was instead showing the effects of CO diffusion out of the cigarette with flow rate (Baker & Crellin, 1977) rather than a measure of inhalation volume. Hee et al. (1995) calculated the amount of CO produced by the cigarette from “the mean volume (mL) of CO per mL of smoke under standardized conditions (ISO 3308) and the total smoke volume drawn by the smoker.” The lowest yield cigarettes would have the highest filter ventilation rates and the flow rate through the tobacco column with standardized machine smoking would be the lowest of all cigarette classes tested. This means that proportionally more CO would have diffused out of the tobacco column for this class (Baker & Crellin, 1977). The actual flow rates measured under human smoking were the highest of all the classes tested. Therefore, proportionally less time would be available for CO diffusion with human smoking of the lowest yield products so there would be a proportionally higher concentration of CO relative to the machine smoking. This means that the error in using the CO concentration obtained from machine smoking would be greatest for the lowest yield products leading to the observed trend in inhalation index.

The results of this study showed no trends in inhalation depths or lung exposure times in habitual smokers smoking their own brand of cigarettes having a broad range of tar yields. In addition, no difference in inhalation tidal ratio was found between smokers of mentholated and unmentholated products, consistent with the broader review of the effect of mentholation on smoking behavior by Werley et al. (2007). We found almost identical post-puff inhalation volumes, lung exposure times, and inhalation tidal ratios in our subjects, a finding in agreement with Tobin and Sackner (1982) and the review by Bernstein (2004). The strongest trend observed (slope p = 0.11) was for inhalation volume versus tar yield, and in this case inhalation volume increased, rather than decreased, as tar yield increased. These findings do not support the “inhalation depth” hypothesis as the causative factor for the increased shift in adenocarcinoma incidence with lower tar yielding cigarettes.
